# 25(OH)D3 improves granulosa cell proliferation and IVF pregnancy outcomes in patients with endometriosis by increasing G2M+S phase cells

**DOI:** 10.1186/s12958-023-01165-8

**Published:** 2023-12-05

**Authors:** Rui Hu, Leilei Li, Lanlan Liang, YuXin Qi, Xiaoling Ma, Yuan Yang

**Affiliations:** 1 Lanzhou University, Lanzhou, Gansu 730000 China; 2 First Clinical Medical School of Lanzhou University, Lanzhou, Gansu 730000 China; 3 First Hospital of Lanzhou University, Lanzhou, Gansu 730000 China; 4 Reproductive Medicine Center of the First Hospital of Lanzhou University Key Laboratory for Reproductive Medicine and Embryo, Lanzhou, Gansu 730000 China

**Keywords:** Endometriosis, Follicular Fluid, Proliferation, Granulosa cell, Cell cycle, 25 (OH) D3

## Abstract

**Background:**

The 25-hydroxyvitamin D3 (25 (OH) D3) is crucial for follicular development. This study aimed to investigate the relationship between the level of 25 (OH) D3 in endometriosis patients, pregnancy outcomes of in vitro fertilization (IVF), and the underlying mechanism.

**Methods:**

The 25 (OH) D3 levels in serum and follicular Fluid (FF) samples were detected using enzyme-linked immunosorbent assay (ELISA). Clinical features and pregnancy outcomes of endometriosis patients were also compared between the deficient group (< 20 ug/ml) and the adequate group (≥ 20 ug/ml). The effects of 25 (OH) D3 on the proliferation and cell cycle of human ovarian granulosa cells were respectively detected by CCK-8 assay and flow cytometry (FCM). The differentially expressed genes (DEGs) in granulosa cells of endometriosis and tubal infertility patients were screened from GEO database. The effects of 25 (OH) D3 on the expressions of CDKN2D, PPARA, TGFB2 and THBD were determined using quantitative reverse transcription polymerase chain reaction (qRT-PCR) and Western blot.

**Results:**

The levels of 25 (OH) D3 in serum and FF samples were decreased in endometriosis patients. The deficient group had fewer embryos that can be transferred, lower quality embryos and lower clinical pregnancy rates. Adequate 25 (OH) D3 levels in FF samples was a protective factor for live birth outcome in endometriosis patients. 25 (OH) D3 enhanced the proliferation capacity of granulosa cells (the concentration of 10 nM was the most significant) and increased the proportion of G2M + S phase cells. The expression of CDKN2D was decreased and TGFB2 and THBD were significantly upregulated.

**Conclusions:**

25 (OH) D3 deficiency may be associated with poor IVF pregnancy outcomes in endometriosis patients. 25 (OH) D3 promotes ovarian granulosa cell proliferation by promoting the ability of cells to divide, and may accelerate cell cycle progression by up-regulating THBD and down-regulating CDKN2D expression.

## Introduction

Endometriosis is a common gynecological disease where the glands and endometrial stromal are planted outside the uterus and mainly occurs in ovary, broad ligament, uterosacral ligament, fallopian tube, sigmoid colon, appendix and pelvis [1]. The incidence rate of endometriosis in women of reproductive age is about 10–15%. Endometriosis is mainly manifested by chronic pelvic pain, dysmenorrhea, and infertility [2]. Endometriosis may impair fertility through peritoneal inflammation, endocrine disturbance, and interference with Follicular Fluid (FF) microenvironment, thus affecting ovarian function and ultimately reducing oocyte quality [1]. The dynamic microenvironment formed by FF is extremely important for oocyte development and ovulation. FF appears as a light yellow and semi viscous liquid. It is mainly formed by endogenous secretions (growth hormone, statin, actin, interleukin, anti-Mullerian hormone, etc.), water, electrolytes (chlorine, calcium and magnesium, etc.) and certain molecules (glucose, lipids, amino acids, etc.) [3]. FF is beneficial to follicle growth and oocyte maturation [4]. Infertility in endometriosis is mainly treated through IVF and surgery. IVF is a widely known assisted reproductive technology (ART). Through a combination of clinical drugs and surgery, professional technicians assist in in vitro fertilization of sperm and eggs. Finally, the cultured embryos are implanted into the uterine cavity. Nonetheless, Patients with infertility Endometriosis treated with IVF have poor pregnancy outcomes. Compared with non-endometriosis patients, the rate of fertilization, embryo implantation and clinical pregnancy is significantly reduced in endometriosis patients [5, 6]. They are also at higher risk of pregnancy complications such as preterm birth, placenta previa, placental abruption and hypertension [7]. Furthermore, the rate of embryo implantation of IVF patients is significantly reduced receiving eggs donated by endometriosis women [8]. Therefore, Endometriosis infertility is associated with impaired oocyte quality.

Vitamin D is mainly stored as 25-hydroxyvitamin D3 (25 (OH) D3) [9]. 25 (OH) D3 affects calcium and phosphorus metabolism, and regulates cellular immunity, differentiation, proliferation and apoptosis. The 25 (OH) D3 is also involved in the regulation of female reproductive functions [10]. Vitamin D acts on the female reproductive system, such as ovaries, endometrium, fallopian tubes and placenta, by binding to receptors [11, 12]. Some studies suggest that vitamin D may be involved in inducing and regulating the synthesis of progesterone, human placental prolactin, FSH receptor gene and human chorionic gonadotropin. Vitamin D also promotes the embryo implantation process, calcium uptake by the placenta, and the immune response of the placenta [13]. vitamin D plays an immunomodulatory role in the inflammatory process of endometriosis. 1,25 (OH) 2D significantly can inhibit interleukin-1β and tumor necrosis factor-α (TNF-α) induced inflammatory responses in human endometriosis stromal cells, and reduce the number of endometriosis stromal cells, thereby improving the symptoms of endometriosis patients [14]. Vitamin D supplementation can significantly improve follicular development and dominant follicular formation in polycystic ovarian syndrome (PCOS) patients [15]. Higher levels of 1,25 (OH) 2D in FF are associated with a higher success rate of IVF in women [16]. In addition, high serum 25 (OH) D3 levels can increase sperm motility [17].

Miyashita M found that the serum 25 (OH) D3 levels were lower in endometriosis patients than in healthy controls. Hartell D et al. found that serum 1,25(OH)2D levels were higher in endometriosis patients than in healthy women [18]. A recent large prospective study found that women with higher plasma 25 (OH) D3 levels had significantly lower rates of endometriosis [19]. And another study showed higher levels of vitamin D in patients with endometriosis [10]. Obviously, there is still controversy about vitamin D levels in patients with endometriosis. This is likely to be related to the staging of endometriosis and may require further study of the relationship between endometriosis classification and vitamin D. Therefore, this study aims to determine the level of vitamin D in people with stage II (mild) endometriosis in the same season.

However, the effects of 25 (OH) D3 levels on pregnancy outcomes in endometriosis patients and the underlying mechanisms are unknown. This study aimed to identify the relationship between 25 (OH) D3 levels in serum and FF with pregnancy outcomes in endometriosis patients. Furthermore, the effects of 25 (OH) D3 on the proliferation and cell cycle progression of human ovarian granulosa cells were explored. In this study, the gene expression data of endometriosis patients and control group were statistically analyzed to screen out the genes with significant expression changes (DEGs). We further screened the crossover genes related to vitamin D metabolism to explore their potential mechanisms. The effect of 25 (OH) D3 on DEGs (CDKN2D, PPARA, TGFB2 and THBD) was also assessed. CDKN2D (P19), a cyclin-dependent kinase inhibitor 2D, causes activation of the p19 signaling pathway, inhibits granulosa cell proliferation, and leads to female infertility [20]. And it can affect the differentiation of blastomeres in 2-cell stage of mice [21]. PPARA, a subtype of peroxisome proliferator-activated receptor, is involved in the regulation of lipid metabolism, inflammation, reproduction and cell growth and differentiation, and plays a role in the maintenance of pregnancy [22]. TGFB2, transforming growth factor-beta 2, one of the major immune and inflammatory factors responsible for regulating cell proliferation, differentiation, angiogenesis, and immune response. Previous studies have found that TGFB is involved in the pathogenesis of endometriosis [23]. THBD, a thrombomodulin that regulates cell proliferation, adhesion, and anti-inflammatory activity, can be detected in granulosa cells of preovulation follicles at elevated levels. In summary, the above genes are closely related to female reproductive function. Therefore, this study may provide a potential intervention reference for improving IVF pregnancy outcomes in patients with endometriosis.

## Materials and methods

### Drugs and reagents

Human Ovary Granules KGN were obtained from Procell Life Science & Technology Co., Ltd (CL-0603, Wuhan, China). KGN cell-specific medium was sourced from Procell Life Science & Technology Co., Ltd (CL-0603, Wuhan, China). 25-hydroxyvitamin D3 (25 (OH) D3) was sourced from Sigma (17938-1MG, Darmstadt, Germany). The short tandem repeat (STR) typing of KGN cell line DNA showed that no cross contamination of human cells was found in the cell line.

### Research population and objects

Endometriosis patients in the First Hospital of Lanzhou University Reproductive Medicine Centre diagnosed between December 2021 and February 2022 were included in the study (75 cases). Patients with infertility due to fallopian tubes in the same period as the control group (219 cases). Inclusion criteria for the endometriosis group: women aged 21 to 38 years undergoing IVF assisted pregnancy; Patients with no history of hormone use within three months; Patients diagnosed with bilateral or unilateral ovarian endometriosis via laparoscopy, laparotomy, and took a biopsy and sent it for pathological examination, with a clear diagnosis [24]. According to the revised American Society of Reproductive Medicine (r-ASRM) classification [25], all patients with endometriosis were classified into stage I (micro): 1–5 points, stage II (mild): 6–15 points, stage III (medium): 16–40 points, and stage IV (severe): > 40 points. All patients with endometriosis included in our study were in stage II. Inclusion criteria for the control group: women aged 21–38 years undergoing IVF; Patients with no history of hormone use within three months; Patients with tubal obstruction (proximal, distal, unilateral, or bilateral) based on hysterosalpingography. Fresh day 3 blastocysts were transferred. To a greater extent, we controlled for confounding factors that affect vitamin D levels (season, stage, ethnic group, diet, medical conditions, kidney and digestive system diseases, etc.). Exclusion criteria included: patients with endometrial polyps, endometritis or endometrial cancer, polycystic ovarian syndrome (PCOS), hyperprolactinemia, hypothyroidism, androgen secreting tumor, Cushing's syndrome, congenital adrenal hyperplasia and diabetes; Patients with congenital abnormal uterine development, diseases that affect calcium or vitamin D anabolism; Patients with systemic conditions that are at high risk of pregnancy complications (hypertension, diabetes, coronary artery, kidney and liver diseases). The study was approved by the Clinical Research and Ethics Committee of the Lanzhou University Affiliated First Hospital, and informed written consent was obtained from all patients (Ethics number: LDYYSZLL2023-09).

### ELISA assay

ELISA assay was conducted as follows: 5 mL fasting venous blood was extracted via venipuncture, mixed upside-down, then left at room temperature for 2 h. The sample was centrifuged at 4000 r/min for 5 min, and the upper serum was frozen at -80 °C. FF was collected after egg extraction, centrifuged at 2000 r/min for 10 min, and the supernatant was frozen at -80 °C. Follicular fluid was collected during egg retrieval, and blood contaminated follicular fluid was not included. An enzyme-linked immunosorbent assay (ELISA) kit (Mei Jing Biotechnology Co., LTD., JM-04677H2, Jiangsu, China) was used to measure 25 (OH) D3 levels. The samples and standard product were reacted at 37 °C for 30 min. The samples were then washed and reacted with enzyme-labeled reagent for 30 min. Finally, the samples were washed, followed by addition of color developing agent A, B. A termination solution was used to stop the reaction. OD value was detected at 450 nm to calculate the actual concentration of the final sample.

### Cell counting kit 8 assay

Normal ovarian granule KGN cell line was used for CCK8 assay. The sample was cultured at 37 °C in 5% CO2 while cell growth was evaluated daily. The medium was changed regularly, and cell passage was performed after 2–3 days. Briefly, 1 × 10^5^ KGN cells (per well) were inoculated in 96-well plates for 24 h, then treated with 25 (OH) D3 (1 nM, 10 nM, 100 nM, 1000 nM) for 0 h, 6 h, 12 h, 24 h. The medium was removed, then the sample was washed with PBS. CCK8 solution (10 μl; coolaber Technology Co., LTD., SK2060-500 T, Beijing, China) was added to each well, then incubated at 37 °C for 4 h. The absorbance was measured at 450 nm with an enzyme marker. Cell viability in each group was calculated as follows: cell viability = [OD (dosing) – OD (blank)] / [OD (no dosing) –OD (blank)] × 100%.

### Flow cytometry

The cells were treated with 25 (OH) D3, then underwent trypsin hydrolysis (0.25% trypsin). The cells (1 × 10^6^) were then washed with PBS, centrifuged at 1000 r/min for 5 min, and supernatant was discarded. DNA Staining solution (1 ml) and 10 ul Permeabilization solution were added to the cells (Linke Biological Co., LTD., CCS012, Hangzhou, China), mixed and incubated in the dark at room temperature for 30 min. Cell cycle was then detected via flow cytometry (BD, LSRFortessa, USA). The ratio of G0G1, S and G2M phase cells was also analyzed. Proliferous index (PI) was calculated as follows: PI = (S + G2M)/(G0G1 + S + G2M).

### Bioinformatics analysis

Granulosa cell expression of patients with ovarian endometriosis and tubal factor infertility was searched from the online website Gene Expression Omnibus (GEO, http://www.ncbi.nlm.nih.gov/geo/). GSE168214 dataset contains three pairs of endometriosis patients and tubal infertility patients with ovarian granulosa cell samples. Herein, the DEGs were screened at threshold | log2FC |≥ 1 and *p* < 0.05. The genes associated with 25 (OH) D3 anabolic metabolism were screened from Gene Set Enrichment Analysis (GSEA) (gsea-msigdb.org). Interactive genes were obtained from DEGs and vitamin D anabolism related genes. The DEGs were analyzed using Gene Ontology (GO) enrichment pathway to explore the functions and action pathways and understand the underlying molecular mechanisms.

### Quantitative real-time PCR

RNA was extracted from cells using TRIzol (Coolaber, Beijing, China), RNA was reverse transcribed to prepare complementary DNA (cDNA). Quantitative real-time PCR was perfumed using fluorescence quantitative premix kit (TIANGEN, Beijing, China). After adding enzyme-free water, primer R, primer F, cDNA and Super Real PreMix Plus to the octet tube, centrifuge instantaneously and set the program and cycle. The mRNA expression levels of CDKN2D, PPARA, TGFB2 and THBD in the treatment group were analyzed using real-time PCR system. GAPDH was used as an internal reference gene. mRNA expression levels were calculated using the 2 − ΔΔCT method (Table 1).

**Table 1:** Genes and primer sequences

Gene	Primer sequence
GAPDH	forward:5’-GGAGCGAGATCCCTCCAAAAT-3’
	reverse:5’-GGCTGTTGTCTACTTCTCATGG-3’
CDKN2D	forward:5’-TCACACTGCTGTGGTCAGCTTT-3’
	reverse:5’-TCACACTGCTGTGGTCAGCTTT-3’
PPARA	forward:5’-GAGTTTATGAGGCCATATTCGCCAT-3’
	reverse:5’-TCACAGAACGGTTTCCTTAGGCTT-3’
TGFB2	forward:5’-GTTCGATTTGACGTCAGCAAT-3’
	reverse:5’-CAATCCGTTGTTCAGGCACTCT-3’
THBD	forward:5’-CCCTGAACAAGAAT TGGAAGCT-3’
	reverse:5’-GGAGCCTAGGATT CTGCATTTCTA-3’

### Western blot assay

KGN cell samples were lysed and centrifuged to extract protein. The lysate was added and sonicated on ice, centrifuged at 12000 r/min for 15 min and the supernatant was removed. The supernatant is the total protein solution. Protein concentration was measured using the Bicinchoninic Acid (BCA) method. The protein was denatured by adding loading buffer and boiling at 100 °C for 15 min, and stored at -80 °C in portions. The sample underwent electrophoresis after gluing at 80 V for 30 min, then at 120 V for 60 min. The sample was transferred to PVDF membrane, then sealed with 5% skim milk for 1 h. The sample was incubated with diluted primary antibody (1:1000, San Ying Biotechnology Co., LTD., 14318–1-AP, 10272–2-AP, 19999–1-AP, 15540–1-AP, 81115–1-RR, Wuhan, China) at 4℃ overnight. The sample was washed thrice the next day using TBST (10 min each wash), then incubated with secondary antibody (1:10000) at room temperature for 1 h. Color development was conducted for gel imaging analysis.

### Data and statistical analysis

SPSS 22.0 and GraphPad Prism 9 were used for all statistical analyses. All experiments were performed in triplicates. Continuous variables were represented by mean ± standard deviation and compared using t-test. If normality was not met, Mann Whitney U test was used for comparison. Quantitative data that did not conform to normal distribution were expressed as median and quartile [M (P25, P75)]. Classification variables were expressed as percentages. Chi-square test was used for the comparison of qualitative data between groups. The influencing factors of pregnancy outcome in endometriosis patients were analyzed via Logistic regression. Pearson correlation analysis was also conducted. *P* < 0.05 was considered statistically significant difference.

## Results

### 25 (OH) D3 levels in serum and FF samples of endometriosis patients

ELISA was used to detect 25 (OH) D3 levels in serum and FF samples of the control and endometriosis groups (Table 2). The 25 (OH) D3 levels in serum and FF samples were significantly lower in endometriosis group (*P* < 0.05).

**Table 2:** 25 (OH) D3 levels in serum and FF of endometriosis and control groups

Group	Control group	Endometriosis group
25(OH) D3 levels in serum(ng/ml)	41.050 ± 54.010	15.688 ± 4.568*
25(OH) D3 levels in FF(ng/ml)	33.570 ± 29.183	15.900 ± 8.783***

25 (OH) D3 in serum and FF samples of endometriosis patients and control patients (ELISA). Data are shown as mean ± standard deviation

*p < 0.05

***p < 0.001

### IVF clinical pregnancy outcomes in endometriosis patients and controls

The general clinical features and pregnancy outcomes of the two groups are shown in Table 3. Compared with the control group, the clinical pregnancy rate was significantly lower in the endometriosis group (*P* < 0.05). The live birth rate was slightly reduced in the endometriosis group.

**Table 3:** Clinical features and pregnancy outcomes of endometriosis group and control group

Group	Control group	Endometriosis group
Age (years)	30.11 ± 3.188	30.84 ± 3.844
BMI(kg/m2)	22.99 ± 2.850	22.67 ± 3.138
History of adverse pregnancy (times)	0.00(0.00,1.00)	0.00(0.00,1.00)
Infertility years (years)	3.00(2.00,5.00)	3.00(2.00,5.00)
Assisted reproduction history (times)	0.00(0.00,1.00)	0.00(0.00,1.00)
FSH(U/L)	6.40(5.40,7.90)	6.30(5.50,8.20)
LH(U/L)	5.00(3.60,7.20)	5.00(3.80,7.00)
E2(pg/mL)	36.60(24.10,54.30)	37.00(23.00,50.20)
AMH(ng/mL)	2.21(1.43,3.20)	1.92(1.33,2.89)
P(ng/mL)	0.22(-0.21,0.44)	0.30(0.12,0.40)
PRL(ng/mL)	18.5(13.40,25.20)	20.00(15.20, 28.90)
Number of eggs harvested	16.00(10.00,23.00)	14.00(10.00,23.00)
MII rate(%)	85.6(3077/3596)	83.4(990/1187)
High quality embryo rate(%)	42.5(491/1154)	37.5(147/392)
Number of transplantable embryos	4.00(3.00,6.00)	5.00(3.00,7.00)
Number of transplanted embryos	1.00(1.00,2.00)	1.00(1.00,2.00)
endometrial thickness at the day of Embryo transfer (mm)	10.00(8.50,11.00)	10.00(9.00,11.00)
Clinical pregnancy rate (%)	71.2(156/219)	58.6(44/75)*
Abortion rate (%)	17.9(28/156)	11.3(5/44)
Ectopic pregnancy rate (%)	3.84(6/156)	4.54(2/44)
Live birth rate (%)	54.7(120/219)	49.3(37/75)

*Abbr*. *BMI* Body Mass index, *FSH* follicle-stimulating hormone, *LH* basic luteinizing hormone, E2, basal estradiol, *AMH* anti-Mullerian hormone, *P* progesterone, *PRL* prolactin; High quality embryo = Grade 1 + Grade 2 embryos. Grade 1 embryos had 6–10 cells, a fragmentation of 0–10%, and perfect symmetry; Grade 2 embryos had either 6–10 cells with fragmentation of 0–25% and moderate symmetry or they had 4–6 or > 10 cells with fragmentation of 0–10% and perfect symmetry. High quality embryo rate = number of (Grade 1 + Grade 2 embryo)/total number of retrieved embryos × 100%. Transferable embryo: The embryo has more than 4 cells, the fragment content is < 25%, the size of most cells is consistent with the developmental stage, and there is no evidence of multiple nuclei. MII rate = MII ovum/ovum harvested × 100%; Clinical pregnancy rate = clinical pregnancy number/transplant cycle number × 100%; Abortion rate = number of abortions/number of clinical pregnancies × 100%; Ectopic pregnancy rate = ectopic pregnancy number/clinical pregnancy number × 100%; Live birth rate = number of live births/transplant cycles × 100%

^*^p < 0.05

### 25 (OH) D3 levels in FF affect embryo quality and IVF pregnancy outcomes in endometriosis patients

The endometriosis patients were divided into deficient group (*n* = 58, < 20 ug/ml) and adequate group (*n* = 17, ≥ 20 ug/ml) based on the 25 (OH) D3 levels in FF samples. The general clinical data, embryo-related conditions and clinical pregnancy outcomes of the two groups are shown in Table 4. Age, BMI, history of adverse pregnancy, Infertility years history of assisted reproduction, FSH, E2, AMH, PRL, P, number of eggs harvested were not significantly different between the two groups. Besides, MII oocyte rate, endometrial thickness at the day of embryo transfer, number of transplanted embryos, abortion rate, ectopic pregnancy rate and live birth rate were not significantly different between the two groups. High quality embryo rate, number of transferable embryos, and clinical pregnancy rate of the insufficient group are lower than those of the adequate group (*P* < 0.05).

**Table 4:** Clinical features and pregnancy outcomes in the deficiency and adequate groups

Group	Sufficient group(≥ 20 ug/ml)	Deficiency group (< 20 ug/ml)
Age (years)	30.59 ± 3.554	30.93 ± 3.957
BMI(kg/m^2^)	22.25 ± 3.436	23.26 ± 4.750
History of adverse pregnancy (times)	1.00(0.00,1.00)	0.00(0.00,1.00)
Infertility years (years)	3.00(2.00,5.50)	4.00(2.00,5.25)
Assisted reproduction history (times)	0.00(0.00,1.00)	1.00(0.00,2.00)
FSH(U/L)	7.2(5.95,8.1)	6.25(5.10, 8.40)
LH(U/L)	5.5(4.25.7.05)	4.75(3.25,7.23)
E2(pg/mL)	34.00(27.00,40.80)	39.25(21.30,51.45)
AMH(ng/mL)	2.03(1.44,3.49)	1.83(1.18,2.81)
P(ng/mL)	0.30(0.20,0.35)	0.30(0.11,0.40)
PRL(ng/mL)	20.00(17.30,29.30)	19.00(14.23,27.35)
Number of eggs harvested	21.3(15.4,26.75)	17.5(11.00,25.05)
MII rate(%)	85.3(284/333)	82.7(706/854)
High quality embryo rate(%)	47.4(55/116)	33.3(92/276)*
Number of transplantable embryos	5.00(5.00,8.5)	4.00(2.75,6.00)*
Number of transplanted embryos	1.00(1.00,2.00)	1.00(1.00,2.00)
Endometrial thickness at the day of embryo transfer (mm)	10.00(9.125,11.750)	10.00(9.00,11.00)
Clinical pregnancy rate (%)	82.4(14/17)	51.7(30/58)*
Abortion rate (%)	14.3(2/14)	6.67(2/30)
Ectopic pregnancy rate (%)	0.00(0/14)	6.67(2/30)
Live birth rate (%)	70.6(12/17)	44.8(26/58)

^*^p < 0.05

### Level of 25 (OH) D3 ≥ 20 ug/ml in FF sample is a protective factor for live birth in endometriosis patients

**Fig. 1:**
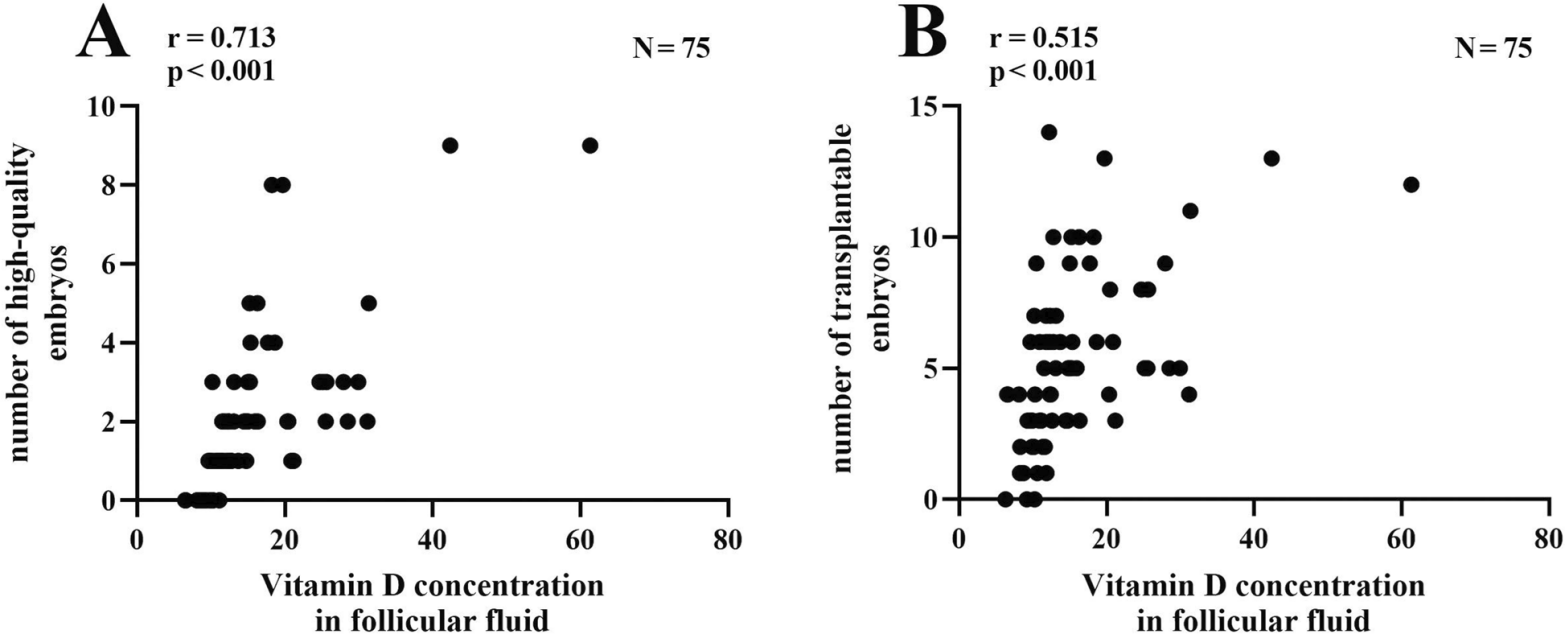
Scatter plot of correlation between 25 (OH) D3 concentration in FF samples with the number of high-quality embryos and transplantable embryos. ***p < 0.001

Logistic regression analysis was performed using live birth as dependent variable (live birth was assigned 0, non-live birth was assigned 1), and 25 (OH) D3 levels of FF, number of high-quality embryos, number of transplantable embryos were as independent variables (Table 5). Results showed that adequate 25 (OH) D3 levels in FF samples were a protective factor for IVF live birth outcomes in endometriosis patients (OR < 1, *P* < 0.05). Furthermore, adequate 25 (OH) D3 levels in FF samples were significantly positively correlated with the number of transplantable embryos and high-quality embryos (*r* > 0, *P* < 0.05; Fig. 1A, B). In addition, the level of 25 (OH) D3 in FF was used as the dependent variable (0 in the sufficient group and 1 in the deficient group), and FSH, LH, E2, AMH, P, PRL were used as the independent variables (Table 6). The results showed that these hormonal indicators did not affect the 25 (OH) D3 levels in FF.

**Table 5:** Logistic regression analysis

Influencing factor	Concrete assignment	B	SE	Waldχ2	P	OR	95%CI
FF 25(OH) D3(ug/ml)	< 20 = 0, ≥ 20 = 1	-1.386	0.645	4.612	0.032	0.250	0.071 ~ 0.886
Number of high-quality embryos	< 2 = 0, ≥ 2 = 1	0.623	0.822	0.574	0.626	0.372	0.372 ~ 9.344
Number of transplantable embryos	< 5 = 0, ≥ 5 = 1	-0.469	0.479	0.958	0.449	0.245	0.245 ~ 1.600

*B* Standardized Coefficient, *SE* Standard Error, *OR* Odds ratio, *95%CI* 95% Confidence Interval

^*^p < 0.05

^**^p < 0.01

^***^p < 0.001

**Table 6:** Logistic regression analysis

Influencing factor	Concrete assignment	B	SE	Waldχ2	P	OR	95%CI
FSH	< 6.3 = 0, ≥ 6.3 = 1	0.187	0.578	0.013	0.908	0.935	(0.301,2.906)
LH	< 5.0 = 0, ≥ 5.0 = 1	-0.426	0.558	0.581	0.446	0.653	(0.219,1.952)
E2	< 37.0 = 0, ≥ 37.0 = 1	0.187	0.552	0.114	0.735	1.205	(0.408,3.559)
AMH	< 1.92 = 0, ≥ 1.92 = 1	-0.285	0.567	0.253	0.615	0.752	(0.247,2.286)
P	< 0.3 = 0, ≥ 0.3 = 1	-0.668	0.594	1.263	0.261	0.513	(0.160,1.643)
PRL	< 20 = 0, ≥ 20 = 1	0.000	0.594	0.000	1.000	1.000	(0.312,3.201)

*B* Standardized Coefficient, *SE* Standard Error, *OR* Odds ratio, *95% CI* 95% Confidence Interval

^*^p < 0.05

^**^p < 0.01

^***^p < 0.001

### 25 (OH) D3 promotes cell proliferation by increasing G2M + S phase ratio of granulosa cells

**Fig. 2:**
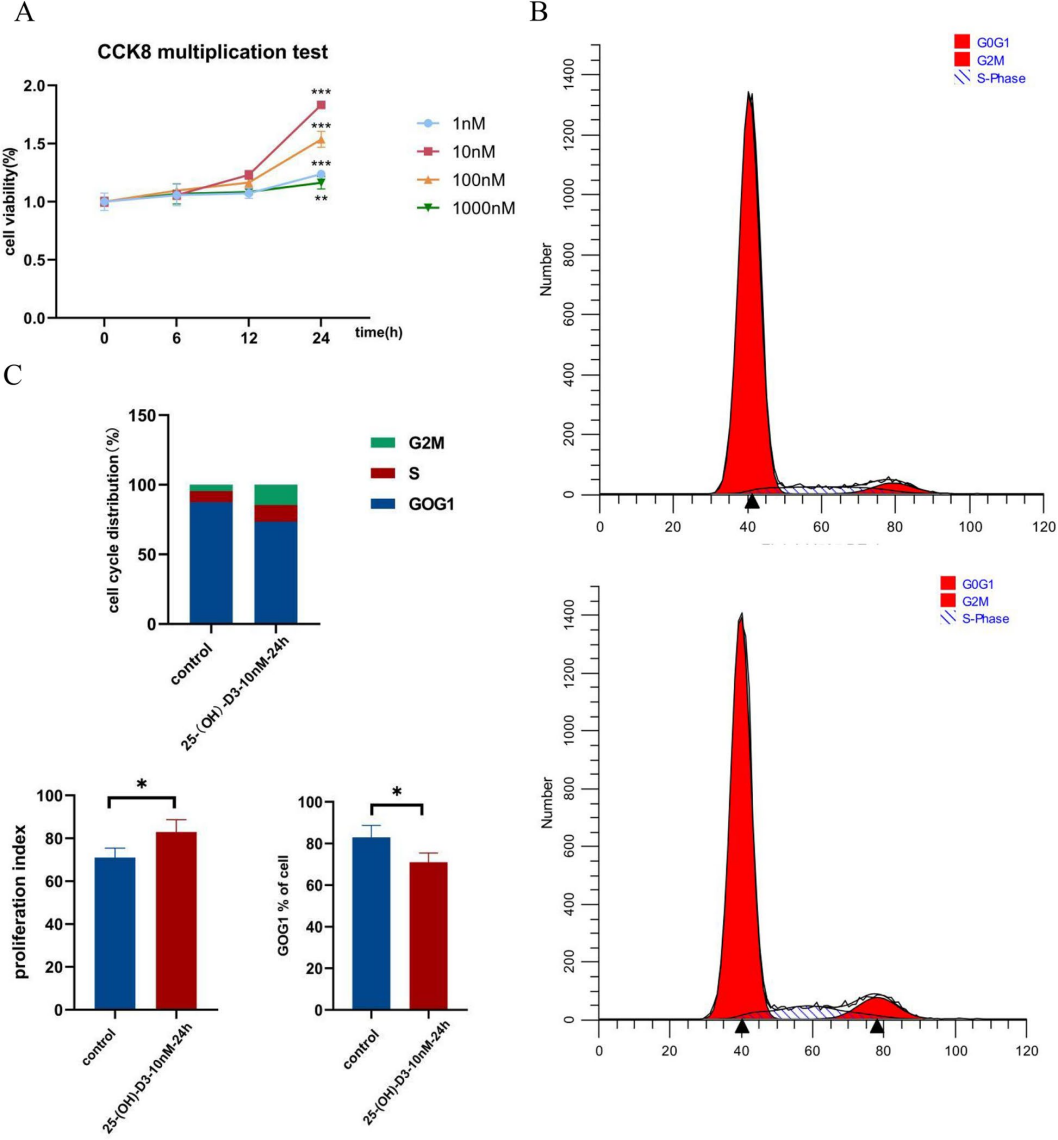
Effects of 25 (OH) D3 on granulosa cell proliferation and cell cycle. **A** Cell viability at different concentrations and time points. **B** KGN cell cycle maps of control group(left) and 25 (OH) D3 treatment group(right). **C** Percentage of G0G1, S, and G2M cell populations in the cycle phase and proliferation index, based on flow cytometry (FCM). *P < 0.05, ***P < 0.001

In this study, cell proliferation was measured by CCK8 assay (Fig. 2A). After 24 h of cultivation with different concentrations of 25 (OH) D3, the cell proliferation activity significantly increased, with the highest at a concentration of 10 nM (*P* < 0.05). Subsequent experiments were conducted at this concentration. Compared with the control group, flow cytometry (Fig. 2B) showed that the percentage of cells in G0G1 phase decreased after 24 h of culture, while the percentage in G2M + S phase significantly increased (*P* < 0.05). Furthermore, 25 (OH) D3 culture increased proliferation index (PI) after 24 h (*P* < 0.05) (Fig. 2C). Also, 25 (OH) D3 reduced G0G1 phase cells, making more KGN cells to enter the active cycle state (G2M + S), thus changing the cycle process and promoting cell proliferation.

### DEGs (CDKN2D, PPARA, TGFB2 and THBD) in granulosa cells of endometriosis patients

**Fig. 3:**
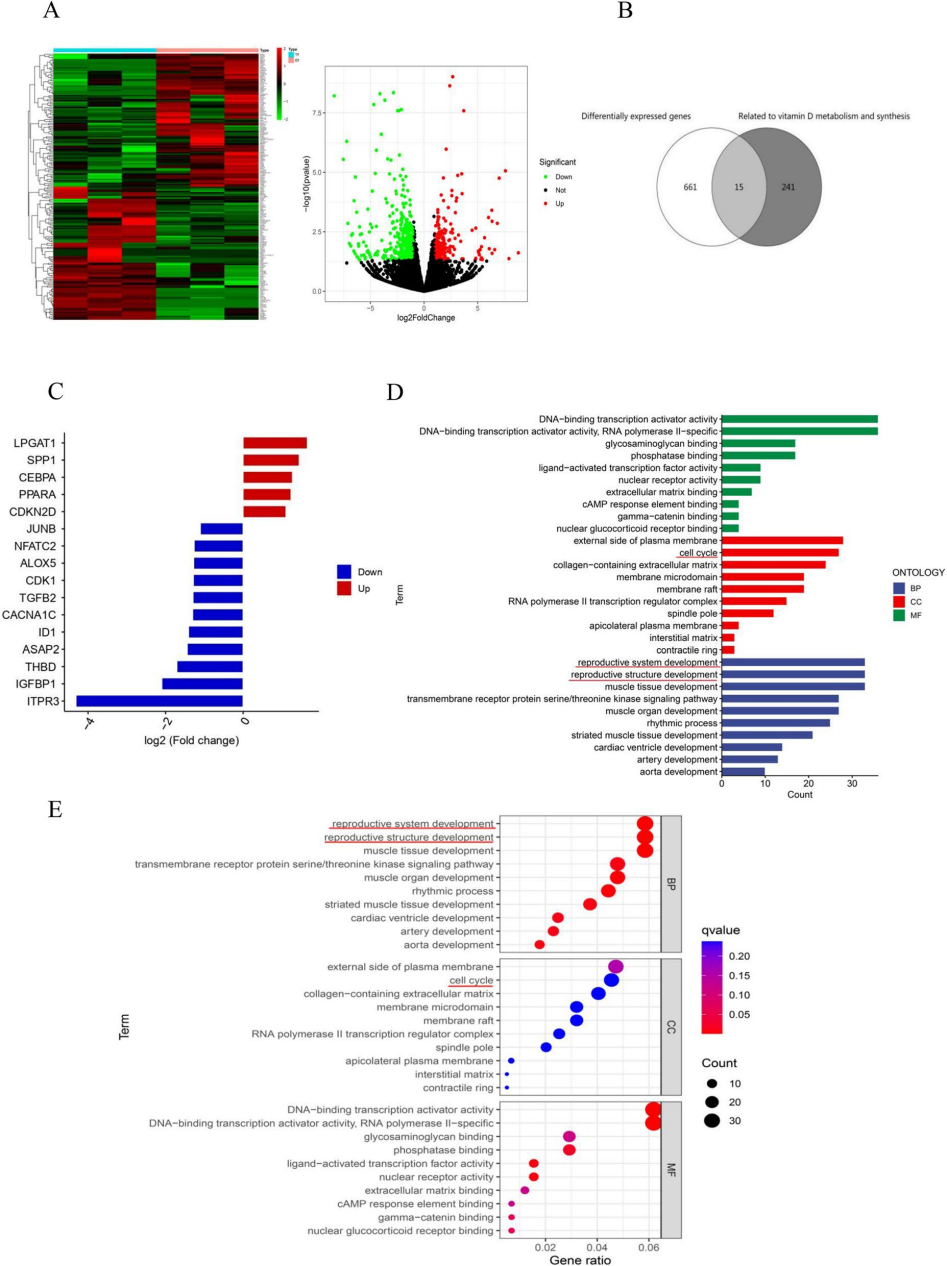
Hierarchical clustering heat maps and volcanic maps of DEGs between endometriosis and control groups. **B** Venn map of overlapping genes. DEGs (white); Genes associated with vitamin D anabolism (dark gray). **C** Cross-gene differential expression level map. Five genes were up-regulated, while the rest of the genes were down-regulated. **D** Functional analysis of DEGs: GEO enrichment column chart: The horizontal axis represents the enriched related pathways; green, red and blue represent molecular function (MF), biological process (BP), and cell component (CC), respectively. The horizontal axis represents the number of enriched differential genes. **E** GO enrichment bubble map: The vertical axis represents the related enriched pathway, while the horizontal axis represents the ratio of gene number enriched in the pathway. The size of the circle represents the number of genes. The larger the bubble, the more genes are enriched. Bubble colors represent statistical significance (the redder the circles, the higher the gene enrichment)

GSE168214 dataset contains ovarian granulosa cell samples from endometriosis patients (*n* = 3) and tubal infertility patients (*n* = 3). Several DEGs (650) were screened (Fig. 3A), of which 222 were up-regulated, and 428 were down-regulated. Among the DEGs and genes affecting 25(OH)D3 anabolism, 15 interacting genes were screened, including ALOX5, TGFB2, ITPR3, NFATC2, SPP1, LPGAT1, ID1, CDKN2D, IGFBP1, CACNA1C, ASAP2, JUNB, THBD, CEBPA, PPARA (Fig. 3B, C, Table 7). GO enrichment analysis showed that the DEGs were related to various pathways, including reproductive structure development, reproductive system development and cell cycle regulation (Fig. 3D, E).

**Table 7:** DEGs

DEGs	Log FC	*P*-val	P-adj	Up/down	Description
ALOX5	-1.275	0.016	0.682	Down	Arachidonate lipoxygenase5
TGFB2	-1.294	0.043	0.954	Down	transforming growth factor-beta 2
ITPR3	-4.311	0.021	0.754	Down	Type 3 inositol 1,4,5-trisphosphate receptor
NFATC2	-1.264	0.039	0.948	Down	nuclear factor of activated T cells 2
SPP1	1.449	0.037	0.939	Up	secreted phosphoprotein 1
LPGAT1	1.657	0.009	0.562	Up	lysophosphatidylglycerol acyltransferase 1
ID1	-1.408	0.002	0.272	Down	Inhibitor of DNA-binding 1
CDKN2D	1.107	0.009	0.562	Up	Cyclin dependent kinase inhibitor 2D
IGFBP1	-2.097	0.001	0.171	Down	Fibroblast growth factor 21
CACNA1C	-1.302	0.0006	0.146	Down	voltage-dependent L-type calcium channel
ASAP2	-1.443	0.008	0.532	Down	PH domain-containing protein 2
JUNB	-1.102	0.021	0.747	Down	AP-1 transcription factor subunit
THBD	-1.709	0.001	0.242	Down	thrombomodulin
CEBPA	1.273	0.016	0.679	Up	CCAAT/enhancer-binding protein-alpha
PPARA	1.237	0.008	0.527	Up	Peroxisome proliferator-activated receptor α

Up/Down: Significantly different genes with up and down regulation

### 25 (OH) D3 can affect the granulosa cell cycle signaling pathway by up-regulating THBD and TGFB2 and down-regulating CDKN2D

**Fig. 4:**
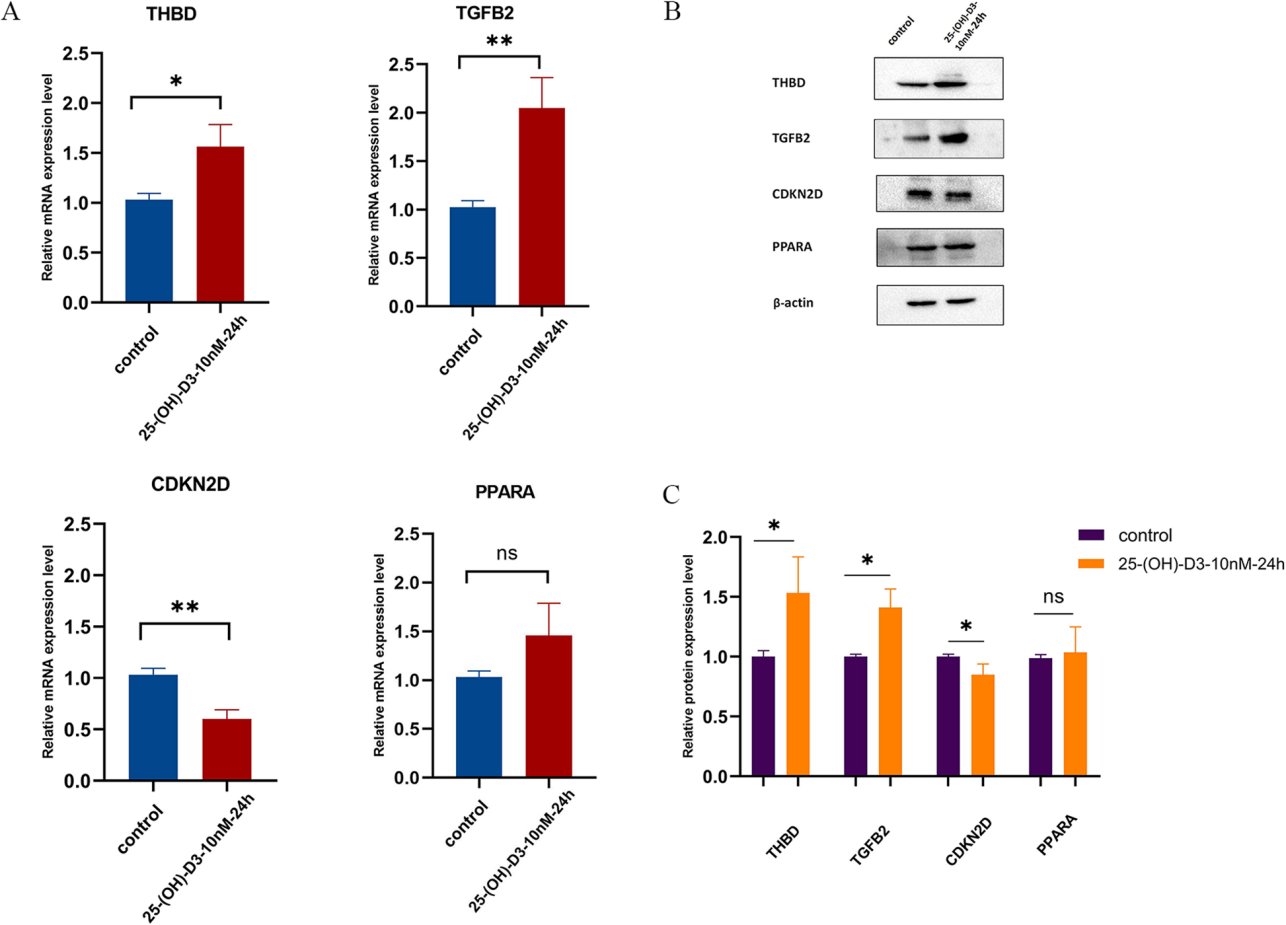
**A** qRT-PCR validation analysis. **B** Protein blotting band map. **C** Protein levels of CDKN2D, PPARA, TGFB2 and THBD in endometriosis and control groups. *P < 0.05

The expression levels of CDKN2D, PPARA, TGFB2 and THBD in granulosa cells were detected by RT-PCR and western blot. Results showed that 25 (OH) D3 treatment did not significantly affect PPARA expression. However, 25 (OH) D3 treatment decreased mRNA and protein levels of CDKN2D, while increasing TGFB2 and THBD levels (both *P* < 0.05) (Fig. 4).

### THBD and CDKN2D expression affect granulosa cell cycle signaling pathways

**Fig. 5:**
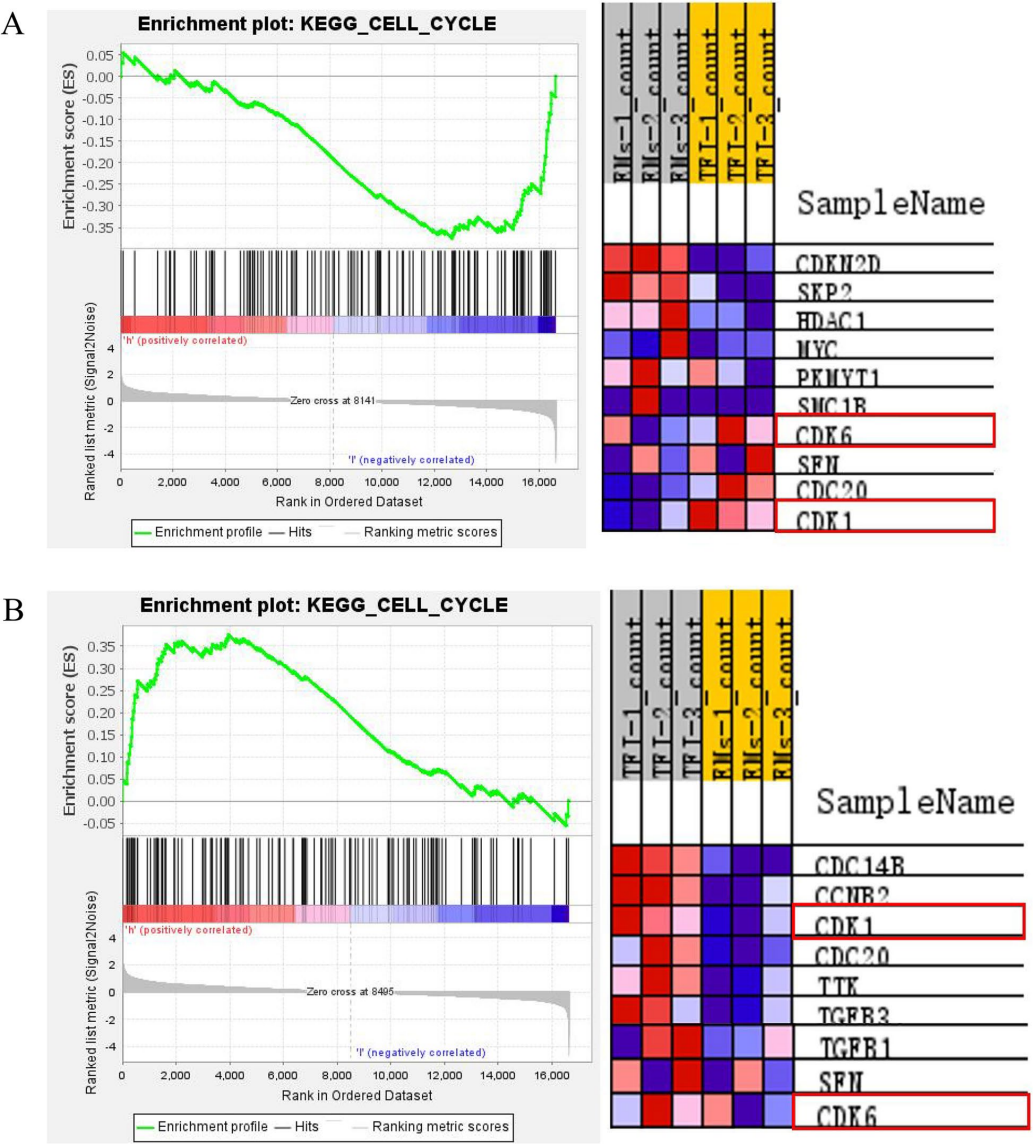
**A** GSEA analysis of CDKN2D and **B** THBD. The horizontal axis represents genes. Pathway enrichment is positively correlated with the genes if the peak value is enriched near "h" (red part). Pathway enrichment is negatively correlated with the gene if the gene is enriched near "l" (in blue). The ones circled in red are significantly enriched core molecules related to cell cycle pathways

GSEA was used to further analyze whether THBD, TGFB2 and CDKN2D affect cell cycle-related pathways (Table 8). Results showed that TGFB2 molecules can affect cell cycle-related pathways (NOM-*p*-val > 0.05). CDKN2D and THBD were significantly enriched in the cell cycle pathway. Results also showed that CDKN2D can down-regulate the cell cycle signaling pathway, while THBD can up-regulate the cell cycle signaling pathway (NOM-*p*-val < 0.05, Table 8) (Fig. 5).

**Table 8:** KEGG analysis

Gene	ES	NES	NOM-*p*-val	FDR-q-val	Up/down
CDKN2D	-0.38	-1.61	0.000*	0.175	Down
TGFB2	0.15	0.70	0.982	1.000	-
THBD	0.38	1.62	0.001*	0.162	Up

^*^P < 0.05

## Discussion

Endometriosis patients account for 25%-40% of the population treated with IVF. However, endometriosis patients have poorer pregnancy outcomes than tubal infertility patients [8]. This study also found that, clinical pregnancy rate was lower in endometriosis patients than in tubal infertility patients. This study aimed to investigate the relationship between 25 (OH) D3 levels and IVF pregnancy outcomes in endometriosis patients and the underlying mechanism, and provided a theoretical basis to improve IVF pregnancy outcomes for further research.

Follicular dysplasia and decreased oocyte quality are associated with poor outcome of IVF treatment in endometriosis patients [6, 26]. Vitamin D levels may be associated with impaired fertility in endometriosis patients [27]. In this study, 25 (OH) D3 levels in serum and FF samples were significantly lower in endometriosis patients than in tubal infertility patients. Previously, vitamin D levels in patients with endometriosis remained controversial. Our results are consistent with those of Ozkan, S et al. [19]. 25 (OH) D3 levels are lower in serum and peritoneal fluid of endometriosis patients [28]. But another study found that the vitamin D levels of these patients increased [10]. This is likely to be closely related to the etiological complexity, stage and seasonal periodicity, duration of sun exposure, regional and ethnic differences [29], Which lead to differences in vitamin D levels. Vitamin D supplementation can significantly relieve pelvic pain symptoms and prevent endometriosis in patients with endometriosis [30–32]. Vitamin D levels were strongly correlated with IVF success (*r* = 0.94), with each nmol/L increase in vitamin D content in FF associated with a 2.4% increase in clinical pregnancy rates [13]. A recent meta-analysis noted that previous studies looked at whether vitamin D supplementation improved pregnancy outcomes in patients with endometriosis, but they did not report r-ASRM staging and whether they had taken additional hormone therapy. Due to heterogeneity and diversity, more research is needed to clarify the role of vitamin D supplements in women with endometriosis [33].

To investigate the effect of vitamin D levels on patients with endometriosis. We grouped the population of endometriosis patients according to 25 (OH) D3 levels. This is novel because it has never been done before. We found that patients with endometriosis who lacked 25 (OH) D3 had significantly lower embryo quality, number of transferable embryos, and clinical pregnancy rate. Ozkan S et al. showed that IVF is associated with higher clinical pregnancy rates in women with higher 25 (OH) D levels in serum and FF samples [34]. In this study, 25 (OH) D3 levels in FF samples were positively correlated with the number of high-quality embryos and transplantable embryos (*r* > 0, *P* < 0.05). Moreover, the live birth rate of endometriosis patients increased with increasing 25 (OH) D3 levels in FF. Halloran B.P et al. also showed that vitamin D deficiency is significantly associated with reduced fertility in female rats [35]. Another study also showed that vitamin D deficiency can reduce mating success and fertility in female rats [36]. High vitamin D levels promote the growth of sinus follicles [37]. Vitamin D level is significantly positively correlated with semen quality [10]. These findings indicate that vitamin D level is directly related to reproductive ability [36]. Our results suggest that sufficient levels of 25 (OH) D3 may play an important role in oocyte development. This study provides new ideas for improving pregnancy outcomes in patients with endometriosis, but its underlying mechanisms are still unclear. Therefore, we have conducted in-depth exploration of the preliminary mechanism. Lack of vitamin D in follicular fluid may be one of the reasons for poor oocyte development.

Throughout the process of maturation and development, oocytes establish a close interaction with the surrounding ovarian granulosa cells, which are arranged in close proximity. The development of follicles primarily relies on the rapid proliferation and biological activity of these granulosa cells [8]. In this study, adding different concentrations of 25(OH)D3 (1 nM,10 nM,100 nM,1000 nM) can significantly promote the activity of granulosa cells after 24 h culture. Xiaolei Yao et al. found that the proliferation rate of goat granulosa cells increased significantly following incubation with vitamin D. Consistent with our results, the proliferation of cells exposed to a concentration of 10 nM was the highest [38]. We also found that 25 (OH) D3 treatment increased the number of G2M + S cells and the proliferation index. More granular cells enter the active state of the cycle, thereby promoting the cell division. Vitamin D influences cell proliferation by regulating the expression of cell cycle-related genes (such as skeletal muscle cells and glomerular mesangial cells, etc.) [39]. Previous studies have also shown that vitamin D3 deficiency results in reduced cell proliferation [40, 41]. Therefore, the low level of 25 (OH) D3 in FF of endometriosis patients alters the expression of genes in the follicular fluid environment and granulosa cells. The transcriptional level of genes in granulosa cells from endometriosis patients was screened to identify differentially expressed genes compared to the control group through KEGG analysis. Several DEGs related to vitamin D, including THBD, TGFB2, PPARA, and CDKN2D, were found to be significantly enriched. These genes are involved in regulating reproductive system development and cell cycle pathways. Afterwards, we analyzed the impact of 25 (OH) D3 on the four target genes.

Treatment with 25 (OH) D3 significantly decreased the expression of CDKN2D mRNA and protein in granulosa cells, while the expression of TGFB2 and THBD was significantly increased. There was no significant change in the expression of PPARA. The enrichment analysis of GSEA cell cycle signaling pathway showed that THBD and CDKN2D were significantly enriched. THBD was associated with up-regulation of cell cycle pathway. The expression of THBD was significantly increased in the granules of preovulation follicles and cumulus cells [42]. THBD has been reported to promote the proliferation of vascular endothelial cells, smooth muscle cell, liver cells, and embryonic trophoblast cells [43–45]. Decreased THBD expression in placenta may lead to increased embryo mortality. In a study conducted by Maskaai et al. [46], it was observed that dendritic cells with up-regulated THBD expression exhibited an increase in the expression of cell cycle genes. The biological function information analysis performed in this study suggested that THBD likely has a positive regulatory effect on cell cycle signaling pathways by up-regulating CDK1, CDK4, and CDK6. CDKN2D was correlated with down-regulate of cell cycle pathway. The activation of the p19 signaling pathway leads to the inhibition of cyclins such as cyclin D1 and CDK4/6. Finally, granulosa cell proliferation is inhibited, leading to female infertility [20]. The study by Hae-ahm et al. showed that CDKN2D knockdown reduced the effect of oxidative stress on the cycle arrest in liver cancer cells and promoted proliferation [47]. Our results observed that reduced expression of CDKN2D reduced cell cycle inhibition. In addition, our results indicate an increased expression of TGFB2, but it is not enriched in the cell cycle signaling pathway. We speculate that it may be involved in other mechanisms and can be further studied.

In summary, endometriosis patients showed low expression level of 25 (OH) D3. The higher the level of 25 (OH) D3, the higher the live birth rate of such patients. Adding 25 (OH) D3 upregulated THBD and downregulated CDKN2D expression. It is likely that this alters the cell cycle-related signaling pathways, and promoted the proliferation ability of granulosa cells. The proliferation and vitality of cells were most significant when cultured at a concentration of 10 nM for 24 h. Limitation of this article: The sample size of patients with endometriosis included in this article is relatively small. This study has potential confounding factors. We conducted education and questionnaire surveys on patients and largely controlled for some confounding factors, such as dietary habits, medical conditions, season, kidney and digestive system diseases, etc. However, potential confounding factors cannot be fully controlled, such as the duration of outdoor sunlight exposure and lifestyle habits. This study found through in vitro cell experiments that vitamin D affects the granulosa cell cycle, but further experiments are needed to verify the potential mechanism, and explore whether there are biochemical or genetic pathways involved in mediation.

**Fig. 6:**
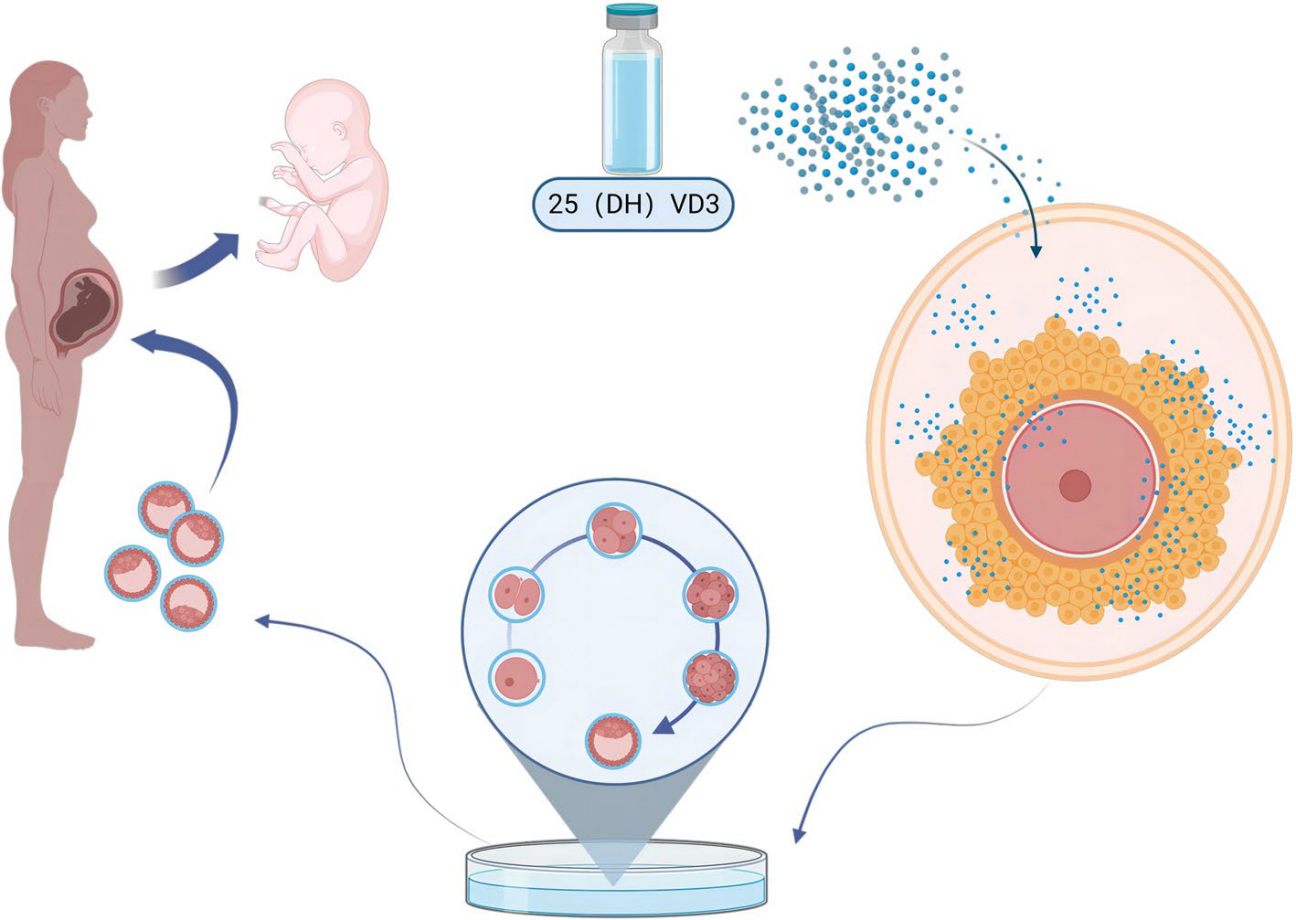
Schematic illustration of the mechanism by which vitamin D improves IVF pregnancy outcomes in endometriosis patients

Finally, in the future, randomized, double-blind, placebo-controlled clinical trials are needed to supplement patients with endometriosis with vitamin D and follow up to observe their IVF pregnancy outcomes (Fig. 6).

## Data Availability

All data are included in this article and its supplementary information files.
